# Parallel Lossless Compression of Raw Bayer Images on FPGA-Based High-Speed Camera

**DOI:** 10.3390/s24206632

**Published:** 2024-10-15

**Authors:** Žan Regoršek, Aleš Gorkič, Andrej Trost

**Affiliations:** 1 Faculty of Electrical Engineering, University of Ljubljana, 1000 Ljubljana, Slovenia; zr1677@student.uni-lj.si; 2 OptoMotive, Mechatronics Ltd., 1000 Ljubljana, Slovenia

**Keywords:** lossless image compression, sensor data processing, parallel processing, high-speed image capture, FPGA

## Abstract

Digital image compression is applied to reduce camera bandwidth and storage requirements, but real-time lossless compression on a high-speed high-resolution camera is a challenging task. The article presents hardware implementation of a Bayer colour filter array lossless image compression algorithm on an FPGA-based camera. The compression algorithm reduces colour and spatial redundancy and employs Golomb–Rice entropy coding. A rule limiting the maximum code length is introduced for the edge cases. The proposed algorithm is based on integer operators for efficient hardware implementation. The algorithm is first verified as a C++ model and later implemented on AMD-Xilinx Zynq UltraScale+ device using VHDL. An effective tree-like pipeline structure is proposed to concatenate codes of compressed pixel data to generate a bitstream representing data of 16 parallel pixels. The proposed parallel compression achieves up to 56% reduction in image size for high-resolution images. Pipelined implementation without any state machine ensures operating frequencies up to 320 MHz. Parallelised operation on 16 pixels effectively increases data throughput to 40 Gbit/s while keeping the total memory requirements low due to real-time processing.

## 1. Introduction

Production quality monitoring, human motion capture in kinesiology [1], image caption of highly dynamic science experiments in welding [2], and vibration analysis of mechanical components [3,4] demand extremely high-resolution image caption at a high frame rate per second (FPS). Our example of professional-grade camera captures 25 mega-pixel images at speeds up to 150 FPS, generating large amount of data [5]. Image compression is applied to reduce channel bandwidth and storage requirements. Numerous different approaches have been undertaken [6], but they can be narrowed down to two types; lossless and lossy. The latter can achieve a higher compression ratio (CR) by dumping some image information and is usually used for web imaging, where a high speed of transfer and low storage capabilities outweigh the lower level of image detail, which is hardly noticeable by the users [7]. Conversely, no original information is discarded in lossless compression. Typical applications for lossless compression are satellite image transfer [8], computer vision algorithms and medical imaging [9], where details are of high importance, such as in wireless capsule endoscopy [10], computer tomography [11], and magnetic resonance imaging [12,13,14].

Typical implementations of image compression are either software based or do not employ source data parallelism. The problem of the former is high latency and low maximum throughput achievable on an embedded CPU, while the disadvantage of the latter is low processing efficiency. High-speed cameras usually employ field programmable gate array (FPGA) chips as an interface to image sensor and on-the-edge image processing. Due to the superiority of FPGA in parallel tasks, we accordingly design our compression algorithm which fully exploits the parallelism from the very beginning on. The image sensor itself generates 128-bit vector representing sixteen consecutive 8-bit pixel values at each clock cycle. Additionally, the algorithm is based on lightweight operations only, which enables it to run at a frequency of up to several hundred megahertz. The high frequency combined with parallelised processing ensures state-of-the-art throughput of the FPGA image compression.

Conventionally, compressed data are clocked out on a single 1-bit output port at an output frequency different from the input frequency. In the usual case of a several-bits-wide input, the output frequency has to be adequately faster than the input one to compensate for the narrower output width, where the exact ratio depends on the bit widths ratio and compression efficiency. This approach is favourable because a simple 1-bit FIFO buffer can be used to collect all bits representing compressed data. However, in the case of high-speed continuous compression, where the input frequency is already very high, it becomes unacceptable since achieving an adequately fast output frequency is impossible. Therefore, the output width must match the input width. The main challenge arises from the fact that the bit-width of the compressed data is not fixed, as it directly depends on the entropy of the input data and varies with each clock cycle. To address this issue, we propose a novel solution with fixed and identical input and output data widths and clock frequencies. We construct a tree-like concatenation structure with one recursive branch. The recursive branch handles the bits that do not align with the 128-bit output width storing them and re-concatenating them with the next set of output data.

**Figure 1:**
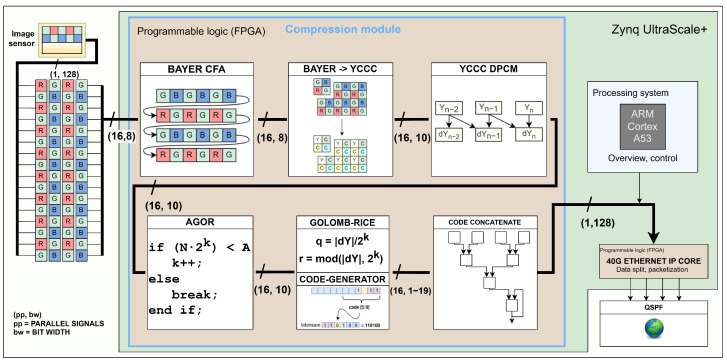
Block diagram of lossless compression on FPGA-based camera.

As illustrated by Figure 1, our compression module interfaces image sensor at the input. At each clock cycle, it receives data in the Bayer colour array [15] for 16 pixels in parallel. Then, every other row is buffered before the red–green–blue channels are translated to *YCCC* colour channels to eliminate colour redundancy. The difference between the neighbouring pixels is then used to remove spatial redundancy. In the next stage, we implement an adaptive Golomb–Rice encoder (AGOR) for entropy coding. Finally, the generated output codes representing the compressed data are concatenated and stored in the output FIFO registers. The output from the compression module is intercepted by the processing system of the Zynq UltraScale+ MPSoC. The header data are extracted and re-packed, while the image data are forwarded to the 40G Ethernet IP core, where packetisation occurs. The core divides the packets across four lanes, each linked to a transceiver capable of a 10 Gbit/s connection, with all lanes connected to the physical QSFP transceiver module.

The goal of this work is to develop and demonstrate a high-throughput hardware implementation of an image compression algorithm capable of interfacing with and exploiting the parallel supply of raw image data directly from the image sensor. The compression component should not be a bottleneck in the system and must not limit the maximum possible throughput, which is governed by the capabilities of the data source and sink modules (i.e., the image sensor and transceiver). To the best of our knowledge, no research has been published on such a parallelised and fully pipelined hardware compression algorithm that meets these requirements.

In our case of high-speed image capture, the proposed compression algorithm can double the streaming frame rate while maintaining the same channel bandwidth and reduce memory consumption by half. Although  image sensors are capable of running at very high frame rates, the maximum frame rate in streaming mode is currently limited by the transmission channel bandwidth. As the data generation rate exceeds the transmission bandwidth, images must be buffered on the camera, which has limited memory resources. Developing such a real-time compression algorithm could increase the maximum streaming frame rate and image storage capacity by reducing the image size. In some cases, image processing is performed on the camera, and only the results are transmitted over the network due to the aforementioned constraints. With the addition of image compression, the images themselves can be attached to the results for diagnostic purposes.

In the next section, we present related work on image compression algorithms and implementations. In Section 3, we explain our solution and present the theory behind it. We then describe the hardware implementation of the algorithm in Section 4. Finally, in Section 5, we show our results and compare our work with others.

## 2. Related Work

There are numerous compression algorithms, but most of them are software-oriented and challenging for efficient hardware implementation in terms of area, power consumption, and maximum achievable frequency. Some conventional compression algorithms are based on complex mathematical operations, such as the discrete cosine transform in JPEG [16] or the discrete wavelet transform in JPEG-2000 [17]. The algorithms, which require large memory blocks and complex operators, are not optimal for an embedded implementation in programmable logic.

The standard for portable network graphics (PNGs) [18] is based on DEFLATE compression [19], Lempel–Ziv–Welch compression and Huffman coding, where the dictionary is built up dynamically [20]. Alternatively, static Huffman coding can also be used, but this one requires a prior analysis of encoding data probability distribution. The problem with such coding schemes is that they rely on a dictionary, which increases memory consumption and the amount of data to be transferred. This is why Golomb–Rice coding has gained acceptance, as it does not require a dictionary, and at the same time offers a competitive compression rate.

An alternative approach to the above-mentioned prediction methods is learned compression methods. In [21], a neural network is trained to learn the probability distribution of pixels, and this knowledge is used for compression. The presented neural network includes 59 K parameters, which occupy a significant amount of the available fast-access memory in a possible implementation on an FPGA device. Furthermore, the compression time is 80 ms for a 64×64 image, which is far too slow for high-speed high-resolution cameras.

A series of articles [22,23,24] have presented the parallel implementation of lossy compression algorithms such as JPEG and DXT on graphic processing units (GPUs). The maximum reported throughput rate is 1385 Mpix/s on an NVIDIA GTX 580 [25] with a typical power consumption of several hundred watts. In this paper, we show that FPGA-based data processing on high-resolution, high-speed cameras [5] can match the throughput of the image sensor for real-time compression while significantly reducing power consumption.

The Consultative Committee for Space Data Systems (CCSDS) has published a CCSDS 123.0-B-1 standard [26] for the lossless compression of multispectral images. It was developed with the aim of hardware implementation that contains only lightweight operations. Nevertheless, a complete implementation covering all possible configuration parameters quickly becomes quite complex. This is the main reason why in the works of [27,28,29], the maximum throughput rates are a few Gbit/s, with the best performance achieved in [30] at 12 Gbit/s when compressing five spectral images in parallel.

Some low-complexity algorithms [31,32,33,34] have been proposed for wireless capsule endoscopy applications, but they generally do not utilise parallelism, which can significantly increase throughput. Other works [35,36] present parallel compression but generate encoding information data, i.e., additional bits to specify whether sections of generated bitstream are encoded or not. In their particular use case of CPU compressed memory, this is appropriate because the encoding bits are immediately available to the decoder, which is implemented in the same hardware as the encoder. However, this cannot be applied to applications where image compression and decompression are spatially and temporally separated and therefore need to be independent of each other. In standard applications for image transmission and storage, the additional coding bits must be included in the transmitted data, which effectively reduces the compression ratio.

Fowers et al. [37] propose an FPGA implementation of a compression algorithm based on the DEFLATE method that exploits the parallelism of input data. Their framework is scalable to 8, 16, 24, and 32 parallel input bytes. Similar to our work, they also face the problem of variable output codeword width. Nevertheless, their implementation turns out to be quite complex, which is why they achieve a maximum frequency of only 175 MHz on an Intel Stratix V FPGA device. Due to parallelism, the throughput is a commendable 22.4Gbit/s with 16 parallel pixels, which is still not enough for some high-speed high-resolution cameras that generate image data at rates of 150/s·5120·5120·8bit=31.5Gbit/s.

The contribution of this work is to bridge the gap in the parallel implementation of real-time image compression while ensuring competitive CR in high-resolution images. An innovative solution is proposed to ensure high throughput compression without additional coding data or a dictionary.

## 3. Proposed Architecture

**Figure 2:**
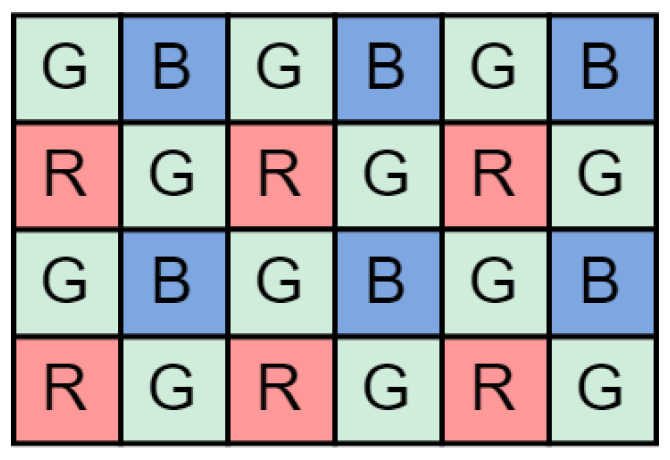
Typical Bayer CFA pattern with twice as many green as red and blue receptors.

A colour image sensor is physically constructed using a *Bayer Colour Filter Array (Bayer CFA)*. Missing colour information for each pixel is interpolated during the demosaicing process [38], where each initial pixel value *R, Gr, B* and *Gb* from the single channel Bayer CFA image is replaced by red, green, and blue channels, effectively expanding the image by a factor of three. An effective approach to image compression is the *compression-first* method, in which the image in its raw Bayer CFA state (Figure 2) is compressed before the demosaicing process. The demosaicing is then performed after decompression on the client side, preventing a threefold expansion of the data before compression begins. In our implementation, the image is compressed by removing colour and spatial redundancy. Finally, the pixel data are entropy encoded using adaptive Golomb–Rice coding, where the coding coefficient *k* is dynamically adjusted based on the data statistics. The parallel output codes are concatenated to form a compressed image bit stream.

Our approach aligns with a broader trend in the literature, where numerous studies have adopted LOCO-I [39] as a baseline framework for improving specific aspects of compression. In this work, the algorithm is modified for better parallelisation and high-throughput implementation on FPGA. Unlike LOCO-I, it does not use context modelling, relying instead on a moving average for parameter *k* estimation. Additionally, it omits the run mode and excludes Huffman coding, which would require additional memory resources. The algorithm is divided into four parts: input data composition, colour transformation, differential pulse code modulation (DPCM) and adaptive Golomb–Rice coding. The colour transformation of the Bayer CFA Red–GreenRed–GreenBlue–Blue (RGrGbB) image into Y-Cd-Cm-Co (YCCC) channels is used to eliminate colour redundancy. Differential pulse code modulation (DPCM) is used to remove spatial redundancy. In the next part, the coding parameters are calculated, and binary codes are generated using adaptive Golomb–Rice coding (AGOR). In the last part, the codes representing the encoded values of 16 parallel pixels are concatenated to generate the final bitstream.

### 3.1. Parallel Pixel Data Supply

**Figure 3:**
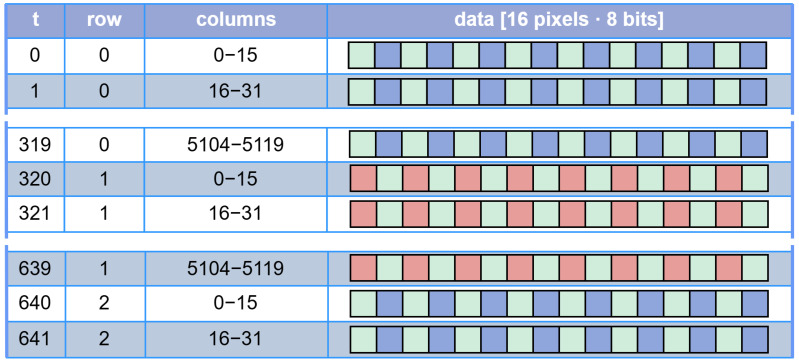
An example sequence of 128-bit sequences representing 16 pixels each, clocked in from the image sensor one after another to the two line buffers. The image is 5120 pixels wide, therefore requiring 5120/16=320 clock cycles to fill one FIFO line buffer.

**Figure 4:**
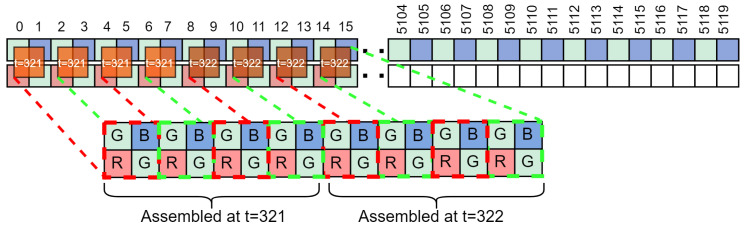
Buffers’ state at t=320. At t=321 and t=322, four *Bayer elements* will be assembled from 16 pixels.

The image sensor interface is commonly implemented in FPGA devices. The high-speed pixel data bus consists of LVDS (Low-Voltage Differential Signalling) connections that provide parallel pixel data. In our case, the data are delivered at each clock cycle from the image sensor as a 128-bit vector representing sixteen 8-bit pixels in a raster scan sequence for a Bayer CFA image as in Figure 3. The next processing stage, colour transformation, requires *Bayer elements* which consist of four neighbouring *R, Gr, B* and *Gb* pixels as illustrated in Figure 4. First, the image sensor supplies complete data for the whole even row (e.g., rows 0 and 2), which consists of *Gb* and *B* pixels only. This even row is therefore fully buffered in memory. Second, data for the entire odd row follow (e.g., rows 1 and 3), which consist of *R* and *Gr* pixels. Once at least 16 pixels for the second row are buffered in memory, the assembly of *Bayer elements* can begin. Therefore, in each clock cycle, four *Bayer elements* are assembled from 16 pixels, with 8 pixel values taken from each row buffer as illustrated in Figure 4. Buffering is realised with FIFO registers. We keep the latency low by performing compression in real time, i.e., as soon as new data are available from the image sensor, they are processed and discarded immediately. Note that in general, the number of pixels compressed in parallel in each clock cycle does not have to be fixed at 16 but is scalable and only limited by the available hardware on the FPGA.

### 3.2. Colour Transformation

**Figure 5:**
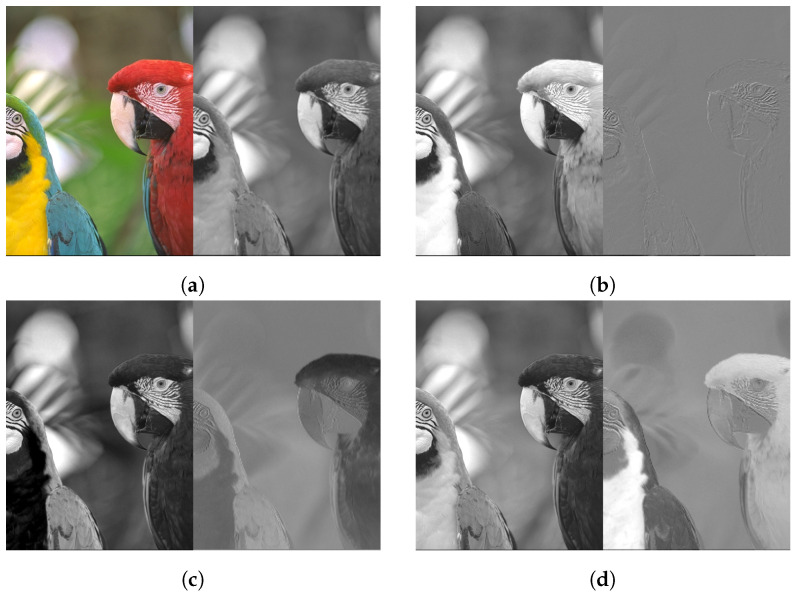
Kodak 13 from KODAK dataset [40]: The content of the image is clearly recognisable in all three RGB colour channels (left). The details are much less pronounced after YCCC transformation (right). (**a**) Colour image and *Y* channel; (**b**) red and *Cd* channels; (**c**) green and *Cm* channels; (**d**) blue and *Co* channels.

Each colour channel in RGB space contains repetitive, i.e., redundant, information as can be seen on left images of Figure 5. The redundant data can be removed by colour conversion to another colour space in which the secondary channels contain much less information as shown on the right images of Figure 5. We use the colour space proposed in [31], wherein the authors analyse different colour spaces that are relatively low in computational cost but still effective in reducing cross-channel correlation. Among several transformations, they find that the *Y-Cd-Cm-Co* colour space achieves the lowest inter-channel correlation, resulting in the highest CR. *Y* corresponds to luminance and is equal to the sum of all four Bayer CFA colour channels. Chrominance information is stored in *Cd*, *Cm*, and *Co* as the difference between the initial Bayer CFA colour channels. We further optimise the translation matrices as defined in (1) and (2). The Bayer CFA to YCCC translation is calculated by addition and subtraction only, which means that no multiplication hardware is used:

(1) $$\left[\begin{array}{cccc} 1 & 1 & 1 & 1\\ 1 & 0 & 0 & -1\\ 1 & -1 & 0 & 0\\ 0 & 1 & -1 & 0 \end{array}\right]\left[\begin{array}{c} Gr\\ R\\ B\\ Gb \end{array}\right]=\left[\begin{array}{c} Y\\ Cd\\ Cm\\ Co \end{array}\right]$$

(2) $$\left[\begin{array}{cccc} 2 & 2 & 4 & 2\\ 2 & 2 & -4 & 2\\ 2 & 2 & -4 & -6\\ 2 & -6 & 4 & 2 \end{array}\right]\left[\begin{array}{c} Y\\ Cd\\ Cm\\ Co \end{array}\right]=\left[\begin{array}{c} Gr\\ R\\ B\\ Gb \end{array}\right]$$

### 3.3. Differential Pulse Code Modulation

The majority of the image content, such as the background and uniform areas, usually contain only a few details. The pixel values are very similar in these areas, so the same information is repeated in several pixels, which is called spatial redundancy. Therefore, it makes sense to predict the next pixel value and then encode only the difference $x_{d}$ between the predicted $x_{p}$ and the actual value *x*. This method is known as differential pulse code modulation (DPCM):

(3) $$x_{d}\left(r,c\right)=x\left(r,c\right)-x_{p}=x\left(r,c\right)-x\left(r,c-1\right)$$

If the predictions are good, the differences are small, and even approach zero. The prediction $x_{p}$ can be based on any combination of previous neighbouring pixels. To keep the complexity and memory requirements low, a single adjacent predecessor is used for prediction in this work. Although pixels from the preceding row could also be used for prediction, this would require buffering the entire preceding row. Additionally, in such a case, interpolation between multiple source pixels would be needed to obtain the final prediction, increasing the overall computational load.

The exception is the first column where predictions are based on the first pixel of the preceding row:

(4) $$x_{d}\left(r,0\right)=x\left(r,0\right)-x\left(r-1,0\right)$$

The other exception is the very first pixel of the image (seed pixel), for which the predicted value is zero:

(5) $$x_{d}\left(0,0\right)=x\left(0,0\right)$$

### 3.4. Golomb–Rice Encoding

**Figure 6:**
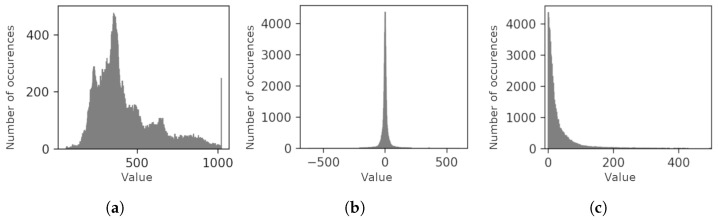
Image data distribution. Initially random distribution (**a**) is transformed by (3)–(5) to Laplace distribution (**b**), and by (6) to exponentially decreasing monotonic distribution (**c**). (**a**): *x*; (**b**): $x_{d}$; (**c**): $x_{pos}$.

The DPCM step converts the random distribution of symbols (represented by an image histogram in Figure 6a into a Laplace distribution that peaks at 0 (Figure 6b). Equation (6) is used to convert difference values to non-negative values to obtain an exponentially decreasing distribution as shown in Figure 6c:

(6) $$x_{pos}=\left\{\begin{array}{cc} 2\cdot |x_{d}| & \mathrm{if}\;x_{d}\geq 0\\ 2\cdot |x_{d}|-1 & \mathrm{if}\;x_{d}<0 \end{array}\right.$$

This new exponentially decreasing distribution fits well with the universal coding systems that are based on such an assumption. They encode smaller values (which also occur more frequently as in Figure 6c) with fewer bits, effectively compressing the information. One of these is Golomb–Rice coding, which is defined in (7) and (8). The value *q* represents a rounded down quotient between the coding value and a constant $2^{k}$. It essentially indicates how often the constant can fit into the coding value. The value *r* is the remainder of the previous integer division, effectively realised with the modulo operator. Both calculations can be implemented cost effectively in hardware using bit shifting (symbolised by ≫), bit slicing (symbolised by [a:b]), and masking operations. The Golomb–Rice code is composed by concatenating *q* consecutive ’1’ bits, the delimiter bit ‘0’, and *r* represented by *k* bits (9):

(7) $$q=\left\lfloor \frac{x_{pos}}{2^{k}}\right\rfloor =x_{pos}\gg k;$$

(8) $$r=mod\left(x_{pos},2^{k}\right)=x_{pos}\left[k-1:0\right]$$

(9) $$G\left(x_{pos},k\right)\equiv \left\{\begin{array}{cc} \underset{\begin{array}{c} \mathrm{quotient}\\ \mathrm{length}\mathrm{of}q \end{array}}{\underset{︸}{111\cdots 11}}0\underset{\begin{array}{c} \mathrm{remainder}\\ \mathrm{length}\mathrm{of}k \end{array}}{\underset{︸}{r_{k-1}r_{k-2}\cdots r_{0}}}, & \mathrm{if}\;q\leq 8\\ \underset{\begin{array}{c} \mathrm{identifier}\\ \mathrm{length}\mathrm{of}8 \end{array}}{\underset{︸}{11111111}}\underset{\begin{array}{c} x_{\mathrm{pos}}\\ \mathrm{length}\mathrm{of}11 \end{array}}{\underset{︸}{x_{10}x_{9}\cdots x_{0}}}, & \mathrm{if}\;q>8 \end{array}\right.$$

### 3.5. Adaptive Golomb–Rice Encoding

**Figure 7:**
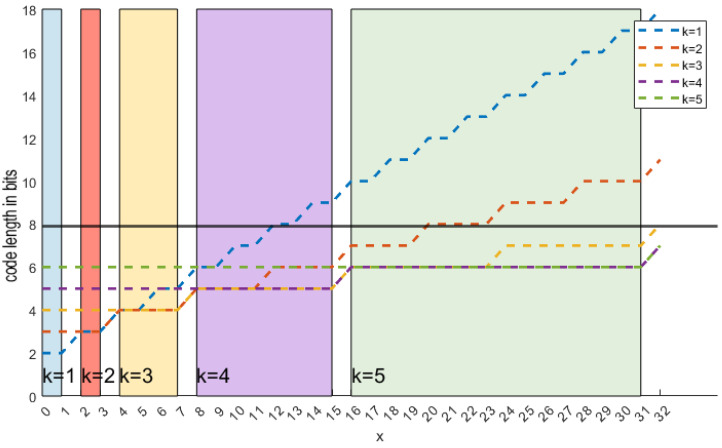
Effect of *k* on code length of Golomb–Rice encoding scheme.

Golomb–Rice coding has a parameter *k* that determines how the code length scales. Since the code is composed of *q* bits, a delimiter bit, and *k* remaining bits, the total length is defined by (10) and shown in Figure 7. A small *k* value is suitable for small values of *x*, while a large *k* is better for large *x*. One is tempted to dynamically adjust the Golomb–Rice encoder (AGOR) based on the value to be encoded. Based on (10), we make two propositions:

(10) $$l\left(G_{\left(x,k\right)}\right)=q+1+k=\left\lfloor \frac{x_{pos}}{2^{k}}\right\rfloor +1+k$$

**Proposition 1.**  *For a given value x from the interval $\left[2^{k_{I}-1},2^{k_{I}}\right)$, choose $k=k_{I}$. Then, the corresponding code $G_{k_{I}}$ is the shortest of all possible codes $G_{k}$.*

**Proposition 2.**  *An encoding scheme based on the rule from the Proposition 1 generates the shortest code for each encoding value $x_{pos}$. The resulting bitstream is therefore the shortest possible and achieves the highest CR theoretically possible as defined by the standard entropy equation.*

However, this *ideally* computed bitstream cannot be decoded due to a problem similar to the *chicken or the egg* paradox; the initially encoded value must be known in order to select *k*, which in turn is needed to decode the initially encoded value. Therefore, in this implementation, the *k* for the $x_{n}$ sample is calculated based on the moving average of the $x_{m}:m<n$ samples as described in Algorithm 1 [39,41]. In the hardware, the first for loop (line 1) is implemented in parallel, while the third for loop (line 8) is pipelined via $k_{max}$ successive modules, each of which contains an *if* statement. The parameter $k_{max}$ is chosen as the largest *k* that still satisfies $q=x_{pos,MAX}/2^{k_{max}}>1$, i.e., the unary part is at least 1 bit wide. Conversely, a case with q=0 means that the value only has to be coded in the remainder of the Golomb–Rice code. This is exploited in the case of a seed pixel by setting the *k* parameter to $k_{seed}=k_{max}+1$, which is 11 for an input width of 8 bits. This effectively results in a seed pixel being encoded in a two’s complement representation.
**Algorithm 1** Dynamically adjustable parameter k. 1:**for** *channel* in *[Y,Cd,Cm,Co]* **do**: 2:    $\left[A_{init},N_{init},k_{init},N_{threshold}\right]\leftarrow \left[32,4,11,8\right]$ 3:    $A\leftarrow A_{init}$ 4:    $N\leftarrow N_{init}$ 5:    $k\leftarrow k_{init}$ 6:    **for** *pixel* in *length(channel)* **do** 7:        k←0 8:        **for** $i=0:k_{max}$ **do** 9:           **if** $2^{k}\cdot N<A$ **then**10:               k←k+111:           **else**12:               k←k13:        $A\leftarrow A+abs(x_{d})$14:        N←N+115:        **if** $N\geq N_{threshold}$ **then**16:           N←N/217:           A←A/2

### 3.6. Code Length Limiting

If $x_{pos}$ is large and parameter *k* is small, quotient *q* from Equation (7) becomes large, which leads to large code length *l* (Equation (10)). This happens in a checkerboard pattern, as *k* becomes very small in uniform areas, while $x_{pos}$ can be large at the black–white border between two squares. Untreated, such an image can generate codes with a width of up to 2040 bits, which means that an originally 8-bit wide pixel code is expanded by more than 250 times. In a system with 16 parallel pixels, the concatenated code could be up to 16,320 bits wide. We solve the problem by limiting the maximum length of the unary parts to 8, resulting in a code defined in the second condition for Equation (9). If the value *q* is greater than eight, the positive value $x_{pos}$ is coded with 11 bits in two’s complement, i.e., we switch from Golomb–Rice coding to two’s complement coding. With this rule, we limit the maximum permissible code length to 19. The identifier for this coding switch is eight consecutive ‘1’s, which are immediately followed by $x_{pos}$ in two’s complement.

### 3.7. Bitstream Generation

Typically, a compression algorithm outputs encoded data by outputting a sequence of bits in a serial *bitstream*. This parallel–serial system can become saturated if the serial output data are not output at a sufficiently high rate, e.g., for a system with 8-bit input data and 1-bit output, the output clock must be at least eight times higher than the input clock. However, in a system with multiple input datasets and a high input frequency, the output frequency cannot be increased appropriately. Instead of increasing the output frequency, the bitstream can also be output in parallel. However, this leads to problems such as assigning a variable-length bitstream to a fixed-width output port, which requires an efficient buffer architecture. The variable length output problem arises from the fact that the algorithm compresses the full-width input code to a narrower representation. The actual amount of compression changes dynamically and depends on the actual data.

The intensity of each pixel is coded with 8 bits in two’s complement. Then, the total width of the input data is P· 8 bit wide, where *P* is the number of pixels supplied in parallel. In our case, we set P=16, but it can easily be parameterised. After compression, the bitstream should be shorter, i.e., consisting of fewer bits than the initial bitstream. This means that each pixel is encoded with fewer than 8 bits on average. While the input to the algorithm is a 128-bit wide input vector representing 16 parallel pixels, the output width is less than 128 bits due to compression and has an unknown length. However, the output port width is fixed at 128 bits to ensure compatibility with other external IPs. It is possible that there are not enough bits (<128) generated for a valid output at every clock cycle due to data compression. On the other hand, even with the code length limiting feature, there can be up to P·19>128 bits generated in a single clock cycle for the edge cases, i.e., a checkerboard. Since other hardware, such as the transmit module, requires fixed length inputs, the output of the compression algorithm must be aligned to 128 bits.

Other works propose the sequential calculation of indices and the sequential concatenation of codes [42]. However, due to the high complexity of the index calculation, these methods lead to a large propagation delay, which considerably limits the maximum achievable frequency, and are only suitable for non-parallelised compression.

## 4. Implementation

**Figure 8:**
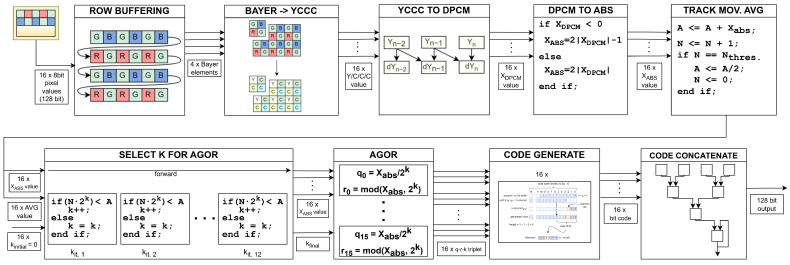
Detailed schematic of complete compression algorithm.

We begin the section with a quick overview of our compression system illustrated in Figure 8. The image sensor supplies image data for 16 pixels as a 128 bit vector. In the *row buffering* module, the first two rows are buffered and *Bayer elements* are assembled. *Bayer to YCCC* takes care of colour redundancy. The next two modules, *yccc to dpcm* and *dpcm to abs*, remove spatial redundancy and shift values to non-negative only. The *track moving average* module keeps track of image statistics used to determine the most optimal compression parameter in *select k for AGOR*. After quotient-remainder-*k* triplets are calculated in *AGOR*, they are supplied to *code generate*, where bit codes for each pixel are generated. Finally, they are merged together into a final fixed-width 128 bit bitstream in *code concatenate*. Each module is pipelined with input interval (II) one. Note that the latency is not one for every module, and the total latency is 78 clock cycles.

The algorithm was first developed and verified in C++ and only then implemented in hardware using VHDL 2008. As the algorithm is designed for low complexity and there are no restrictions on efficiency and parallelism in the software model, the C++ implementation is relatively simple. In the hardware implementation, however, additional requirements were added with regard to memory consumption, parallelism, low propagation delay (propagation delay as a function of the complexity of a subalgorithm), dynamically adjustable ranges, the generation and concatenation of vectors of variable length, and finally the output of a bitstream of variable length via an output port with a fixed width.

The algorithm from the C++ code can be used with high-level synthesis (HLS) tools to generate a VHDL circuit model. However, substantial modifications to our C++ code would be necessary to achieve optimal synthesis results. The unpredictability of HLS hardware implementation could negatively impact the compression throughput and make debugging on the FPGA significantly more challenging. Additionally, since the HLS solution may consume excessive FPGA resources [43], we have decided to use VHDL.

### 4.1. Parallelism

**Figure 9:**
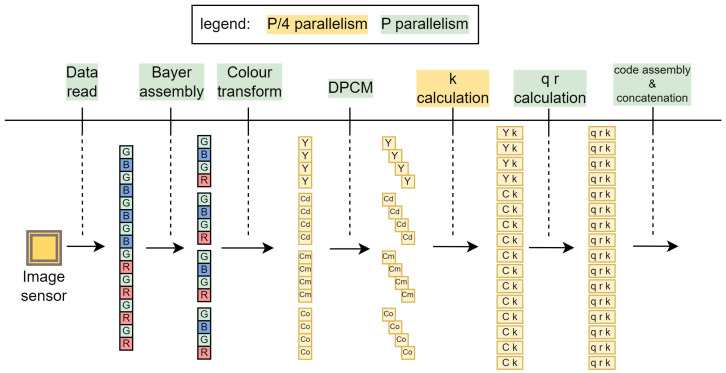
Example of parallelism in case of P=16 parallel pixels supplied by the image sensor.

The main contribution of this work in the field of image compression is the efficient parallelisation of the algorithm. We use partial parallelism in one phase and full parallelism in all other phases as shown in Figure 9. At the beginning, the pixels *P* supplied by the image sensor are read completely independently and in parallel. After the line memories are sufficiently filled, $\frac{P}{4}$ Bayer elements are formed in parallel, and the translation to YCdCmCo is performed. The calculation of the parameter *k* is the only partially parallelised step, where $\frac{P}{4}$ modules are used in parallel to calculate inherently sequential variables from Algorithm 1 as explained in Section 4.2. All other modules are fully parallel with *P* parallel modules.

### 4.2. Dynamically Adjustable Compression Parameter *k*

**Figure 10:**
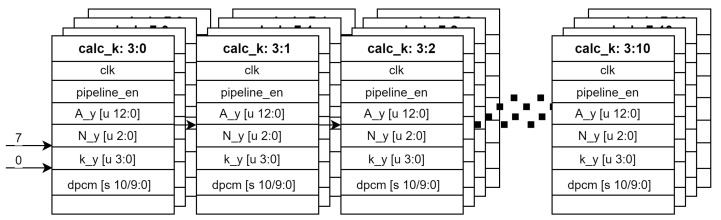
Calculation of *k* as a pipeline. The same pipeline is implemented in parallel for 4 channels.

As described in Algorithm 1, the variables are updated after each pixel (line 6 of Algorithm 1), and the current value of $A_{n}$ depends on the previous value $A_{n-1}$ (line 13). The calculation is sequential by nature, which is not compatible with the fact that the image sensor provides data for multiple pixels in parallel. However, as long as this sequential calculation takes place within one period of the clock, there is no problem. You can mitigate this problem by splitting the algorithm into smaller and shorter calculations, i.e., by implementing a pipeline as we did for the third loop (line 8). We divide the calculation of *k* into several stages as shown in Figure 10. Since the four channels (Y,Cd,Cm,Co) are independent of each other, the pipeline can be implemented partially in parallel.

### 4.3. Code Generation

**Figure 11:**
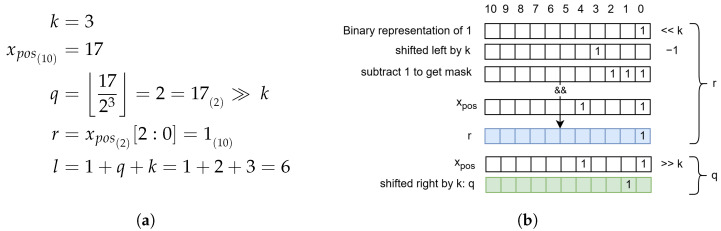
Illustration of calculating *q* and *r* in C++ (**a**) and VHDL (**b**). (**a**) Example calculation of Golomb–Rice quotient and remainder; (**b**) hardware calculation of *q* (green) and *r* (blue) by bit-slicing and bit-shifting. In this case, $x_{pos}=17$, k=3.

In contrast to the classical software (sequential) procedure as in Figure 11a, the code in our hardware implementation is generated in two cycles. In the first cycle, remainder *r* and quotient *q* are calculated, while the actual code is generated in the second cycle. With the bit slicing operator [a:b], a and b are dynamic in our algorithm, i.e., they change depending on the image data entered. Since VHDL does not support dynamic ranges, the bit slice operation is implemented as bit masking and bit shifting instead. As shown in Figure 11b, a unary mask of width *k* is generated for the binary representation of *r*, effectively truncating higher bits. The quotient *q* is simply calculated by bit shift, which is supported in VHDL even with dynamic shift values.

**Figure 12:**
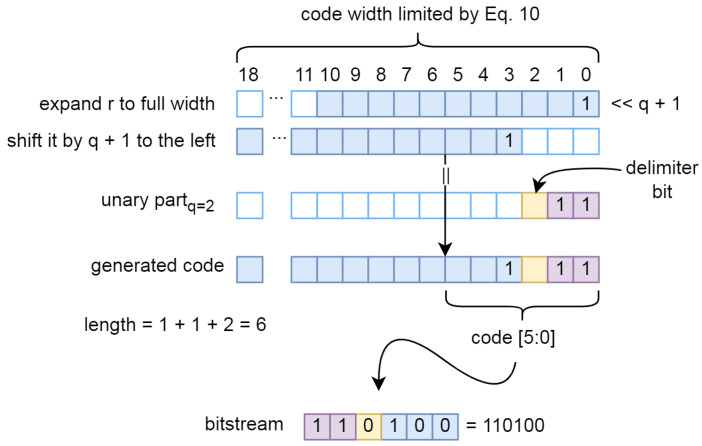
Generation of the final code.

Theoretically, the code is composed by concatenating the unary part, the delimiter bit and the remainder bits. A simple solution would be to use the VHDL concatenation operator &, but the dynamic nature of the problem requires a slightly more complex solution as shown in Figure 12. The final code is generated by concatenating the pre-generated unary part and the *r* shifted to the right by q+1. The addition +1 is added to create space for a delimiter bit ‘0’. At the same time, the code length is calculated to indicate the actual number of valid bits in the fixed-width output that represents the code. The output of the code generation module is therefore a fixed-width vector, with the LSB bits representing the code and the fixed-width vector indicating the number of relevant bits.

### 4.4. Parallel Code Concatenation

**Figure 13:**
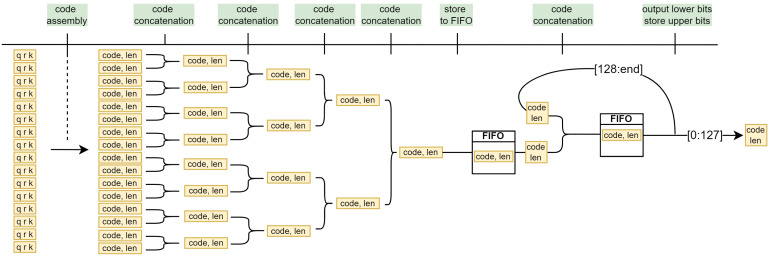
Pipelined bitstream generation on 16 parallel pixels.

Following the discussion in Section 3.7, we propose an extremely simple tree-like solution with a recursive terminal branch as illustrated in Figure 13. In the first stage, 16 parallel code generator modules construct the codes, which are then concatenated in the subsequent layers. The maximum code length in the last layer can be up to 16·19=304 bits wide, which is still a manageable width for a multiplexer. If more than 128 bits are generated, they are output over the next $\left\lfloor \frac{P\cdot 19}{128}\right\rfloor =\left\lfloor \frac{16\cdot 19}{128}\right\rfloor =2$ cycles, and a FIFO module serves as a buffer for the codes generated during this time. Only the lower 128 bits are output at the output for each clock cycle. The remaining bits form new code, which is fed back through a recursive branch. The final output is therefore always a 128-bit wide bit stream with an additional 1-bit valid flag, which indicates that the data on the rail are valid and can be retrieved by the external modules.

## 5. Results

The proposed lossless compression algorithm and its FPGA implementation are presented in terms of compression efficiency, speed and FPGA resource utilisation. Motivated by other works in the field, we evaluate the algorithm with the KODAK dataset [40], the Image Compression Benchmark dataset [44] and our own high-resolution images, which we refer to as the *car* dataset.

To evaluate the compression efficiency, we use the metrics *bits per pixel* (BPP) and *compression ratio* (CR), which are defined as follows:

(11) $$\begin{array}{cc} BPP & =\frac{\mathrm{number}\;\mathrm{of}\;\mathrm{bits}}{\mathrm{number}\;\mathrm{of}\;\mathrm{pixels}} \end{array}$$

(12) $$\begin{array}{cc} CR & =\frac{\mathrm{uncompressed}\;\mathrm{size}}{\mathrm{compressed}\;\mathrm{size}}=\frac{BPP_{initial}}{BPP}=\frac{8}{BPP} \end{array}$$

To evaluate the throughput, we define the absolute or *effective* throughput (13). For a fair comparison, we scale the effective throughput with the number of parallel pixels *P* (14):

(13) $$\begin{array}{cc} \mathrm{EX}\;=\;\mathrm{Effective}\;\mathrm{throughput} & =\mathrm{input}\;\mathrm{data}\;\mathrm{bits}\cdot \mathrm{frequency}\left[\frac{bit}{s}\right] \end{array}$$

(14) $$\begin{array}{cc} \mathrm{NX}\;=\;\mathrm{Normalised}\;\mathrm{throughput} & =\frac{\mathrm{input}\;\mathrm{data}\;\mathrm{bits}\cdot \mathrm{frequency}}{P}\left[\frac{bit}{s\cdot pixel}\right] \end{array}$$

### 5.1. Compression Efficiency

The algorithm works with Bayer CFA pattern images; therefore, RGB colour images are first converted into Bayer CFA patterns during evaluation. This is performed by subsampling the colour image, whereby only one colour component is retained for a single pixel, resulting in a Bayer CFA pattern. The size of the reference file is then defined as the size of the Bayer CFA image, i.e., the number of pixels multiplied by 8 bits (1 byte).

**Figure 14:**
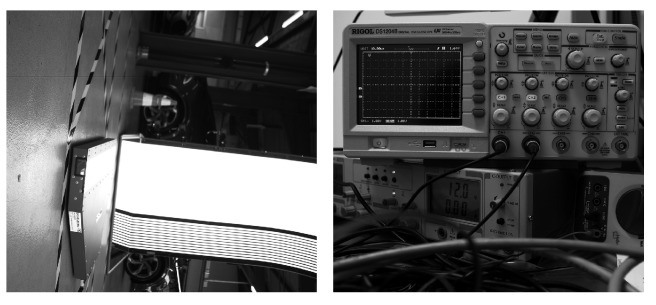
The 18 megapixel images car_01.png and oscilloscope.png with CR 2.26 and 1.80, respectively.

We compare our work with established lossless methods such as PNG and JPEG-LS [45], and more recent work LOCO-ANS [46] on our high-resolution images (18 megapixels, Figure 14). We achieve CR of 2.26 (Table 1), which is due to a sequence of very similar neighbouring pixels, resulting in a large number of difference values close to 0, and requiring only a single bit to encode each pixel. For future evaluation of our work, we run the compression on the Image Compression Benchmark dataset [44] on RGB 8-bit images as in Table 2. Note that we first subsample the images to obtain the Bayer CFA image.

The most established image dataset in the field of hardware image compression is the KODAK image set [40] consisting of 24,768 × 512 images in Table 3. The lower CR value (1.34 on average) is due to the lower resolution and high level of detail of the KODAK images, where neighbouring pixels are less similar. We compare our algorithm with the PNG standard and the works of [32,33,34]. We achieve comparable results to the PNG standard but worse CR when compared to the mentioned works. However, the goal of the proposed algorithm is not a state-of-the-art CR but high throughput with satisfactory CR for high-resolution images.

### 5.2. Maximum Frequency and Resource Utilisation

The algorithm is implemented on Xilinx Zynq UltraScale+ [47] FPGA and runs at 320 MHz. It consumes 14.1k LUT, 14.8 k registers, and 9.5 BRAM blocks which is less than 10% of all available resources, as shown in Table 4. Due to pipelining and parallelism, the effective throughput is continuously 16 pixels per clock cycle, i.e., 16 · 8 bit · 320 MHz = 40.95 Gbit/s. This is equal to 5120 Mpixel/s or 3.61 ms per 18 Mixel image (4512 × 4096). We outperform other FPGA implementations such as [30,37,48] in both effective and normalised throughput. The latter achieves high throughput through a high degree of parallelism like our work. However, our work operates at almost twice the frequency, which increases the effective throughput by the same ratio.

Some of the published works on hardware image compression implement the algorithm on application-specific integrated circuits (ASICs). The comparison of FPGA and ASIC implementations is challenging due to the different technology performance. However, to show the effectiveness of our design, we compare our work with several ASIC implementations. In the works of [31,32,34], the maximum operating frequencies achieved are 250 MHz and 200 MHz, respectively. The effective throughput is not reported, but due to the lack of parallelism, it is estimated to be 1 pixel per clock cycle, which is 16 times less than our work.

The only algorithm that outperforms ours is [36], where the maximum operating frequency for the ASIC design is 370 MHz and the effective throughput is 192 Gbit/s for the parallel processing of 64 pixels. It should be noted that ASICs achieve much higher frequencies than FPGA implementations but at the cost of high development costs and non-reconfigurability. They are usually used in the final stage of a product development after the design has been verified on an FPGA.

## 6. Discussion

Even though we are not focused on achieving a state-of-the-art compression ratio, our results are on par with other well-established compression methods. We speculate that these average CR results are due a single-seed prediction filter besides a generally extremely low complex compression algorithm. The maximum achieved compression ratio is, on average, 2.01 on our *car* dataset, which is equal to 50% image size reduction.

Our goal is to achieve state-of-the-art image compression throughput rate through a parallelised and pipelined design, eliminating the compression part as a bottleneck in the image capture and transfer. We outperform all existing work by a significant margin. With our compression rate achieving 40.10 Gbit/s, we outperform the second-best implementation [37] (22.4 Gbit/s) by 79.0%. We only fall short behind one ASIC-based implementation [36], but this comparison is not apples to apples. In contrast to [46], all parts of our algorithm constantly operate at 320 MHz, which shows how well optimised our implementation is.

## 7. Conclusions

The goal of the research is to fill in the gap in parallel high-speed image compression on FPGA. Several works in this area offer some commendable solutions, but none can match the throughput of modern high-speed high-resolution cameras. We have successfully combined, modified and optimised well-known principles of lossless compression to develop a lightweight, scalable, and high-throughput image compression that can easily keep up with current and future cameras while ensuring a competitive compression ratio. Through effective pipelining, an operating frequency of up to 320 MHz with a continuous throughput of 40.10 Gbit/s is achieved, which significantly outperforms all other existing FPGA-based implementations for image compression. The compression principles presented in this work can also be applied to other compression tasks such as video, audio, or data compression with minimal modifications, and lossy support can be added immediately. Future work will focus more on improving the compression ratio, e.g., by exploring new colour space translations, applying different compression principles and trying out different prediction filters, and possibly extending the architecture to support video compression based on inter-frame prediction.

## Figures and Tables

**Table 1:** CR (BPP in brackets) for lossless compression on 18 Mpixel images. All images are 8 bits-per-pixel initially. Higher CR is better. **Best results**, second-best results.

Image File	PNG (Mode = 9)	JPEG-LS [45]	LOCO-ANS [46]	Ours
car_02.png	1.43 (5.33)	2.00 (4.23)	**2.07 (3.98)**	1.98 (4.05)
car_01.png	1.50 (5.59)	1.89 (4.00)	2.01 (3.86)	**2.05 (3.91)**
car_03.png	1.37 (5.84)	2.03 (3.94)	**2.09 (3.83)**	1.94 (4.12)
car_04.png	1.57 (5.10)	1.98 (4.04)	2.16 (3.70)	**2.26 (3.54)**
oscilloscope.png	1.24 (6.45)	1.49 (5.37)	1.54 (5.19)	**1.80 (4.44)**
Average	1.42 (5.63)	1.88 (4.26)	1.97 (4.05)	**2.01 (3.99)**

**Table 2:** CR (BPP in brackets) for lossless compression on high-resolution images (ICB [44], RGB 8 bit). Higher CR is better. **Best results**, second-best results.

Image File	PNG (Mode = 9)	JPEG-LS [45]	LOCO-ANS [46]	Ours
artificial.ppm	**6.11 (1.31)**	2.40 (3.33)	2.69 (2.97)	2.70 (2.96)
big_building.ppm	1.36 (5.89)	1.45 (5.53)	**1.54 (5.21)**	1.43 (5.61)
big_tree.ppm	1.22 (6.54)	1.29 (6.20)	**1.33 (6.03)**	1.31 (6.10)
bridge.ppm	1.25 (6.40)	1.32 (6.04)	**1.42 (5.64)**	1.29 (6.20)
cathedral.ppm	1.47 (5.44)	1.56 (5.13)	**1.63 (4.92)**	1.39 (5.75)
deer.ppm	1.29 (6.22)	1.35 (5.92)	**1.39 (5.74)**	1.26 (6.33)
fireworks.ppm	2.77 (2.89)	3.16 (2.53)	**3.46 (2.31)**	2.41 (3.32)
flower_foveon.ppm	1.41 (5.67)	1.41 (5.67)	1.43 (5.59)	**2.17 (3.68)**
hdr.ppm	1.43 (5.60)	1.52 (5.25)	1.69 (4.72)	**2.03 (3.95)**
leaves_iso_1600.ppm	1.23 (6.50)	1.23 (6.50)	**1.26 (6.33)**	1.22 (6.54)
leaves_iso_200.ppm	**1.31 (6.11)**	1.25 (6.42	1.26 (6.34)	**1.31 (6.11)**
nightshot_iso_100.ppm	1.55 (5.15)	1.88 (4.26)	**1.98 (4.04)**	1.96 (4.08)
nightshot_iso_1600.ppm	1.32 (6.05)	1.42 (5.65)	**1.47 (5.44)**	1.37 (5.83)
spider_web.ppm	1.60 (4.99)	1.97 (4.07)	2.09 (3.83)	**2.16 (3.70)**
Average CR	1.50 (5.34)	1.54 (5.18)	**1.62 (4.94)**	1.60 (5.01)

**Table 3:** Comparison to other works on KODAK dataset [BPP].

idx	PNG	YLMN [32]	CMBP [33]	[34]	[31]	This Work
1	6.47	6.86	5.48	6.31	7.88	7.11
2	6.42	5.60	4.33	4.93	6.36	5.79
3	6.00	4.95	3.75	4.54	5.84	5.15
4	6.52	5.62	4.38	5.09	6.45	5.79
5	6.55	7.06	5.41	6.34	7.97	7.17
6	5.99	5.84	4.88	5.60	6.74	5.98
7	6.08	5.31	3.96	4.93	6.16	5.48
8	6.49	7.15	5.57	6.67	8.12	7.45
9	5.70	5.41	4.19	4.88	6.22	5.62
10	5.78	5.56	4.23	4.99	6.35	5.74
11	5.94	5.88	4.68	5.42	6.77	6.10
12	6.06	5.19	4.09	4.82	5.99	5.35
13	6.74	7.22	6.14	6.71	8.17	7.37
14	6.42	6.40	5.17	5.86	7.25	6.52
15	6.39	5.56	4.10	4.87	6.43	5.91
16	5.45	5.25	4.38	5.06	6.12	5.42
17	5.30	5.65	4.29	5.10	6.45	5.83
18	6.48	6.50	5.28	5.88	7.36	6.64
19	6.17	5.96	4.71	5.48	6.84	6.14
20	4.20	4.67	3.54	4.60	5.91	4.77
21	5.95	5.74	4.80	5.35	6.56	5.89
22	6.41	6.10	4.85	5.49	6.96	6.29
23	7.03	5.09	3.85	4.51	5.86	5.24
24	5.95	6.49	4.87	5.79	7.41	6.75
AVG	6.10	5.88	4.62	5.39	6.76	6.06

**Table 4:** Resource and throughput comparison to other works. Publications marked with asterisk * are ASIC based.

	[34] *	[31] *	[32] *	[36] *	[43]	[48]	[46]	[30]	[37]	Our
Year published	2019	2018	2017	2019	2023	2019	2021	2019	2015	2024
Parallel pixels	1	1	1	64	1	1	2	5	16	16
Freq (MHz)	200	250	250	370	107	214	87/447	150	175	320
Gate count (k)	4.8	5.2	3.78	83	-	-	-	-	-	-
Memory (k)	10.2	5.74	2.93	-	-	-	-	-	-	-
Registers	-	-	-	-	0.39 k	-	4.2 k	12.8 k	-	14.1 k
LUT	-	-	-	-	1.3	-	4.5 k	14.7 k	-	14.8 k
BRAM	-	-	-	-	4	-	21	37	-	9.5
DSP	-	-	-	-	4	-	2	-	-	-
EX (Gbit/s)	1.60	≤2	≤2	192.00	0.17	0.43	1.98	12.00	22.40	40.10
NX (Gbit/s/pixel)	1.60	≤2.00	≤2.00	3.00	0.17	0.43	0.99	2.40	1.40	2.56

## Data Availability

Source code for C++ implementation of encoder and decoder will be available after publication at https://github.com/joej970/OMLS (accessed on 8 August 2024).

## Abbreviations

The following abbreviations are used in this manuscript:

AGOR	Adaptive Golomb–Rice Encoder
ASIC	Application-Specific Integrated Circuit
BPP	Bits Per Pixel
C++	C Plus Plus
CCSDS	Consultative Committee for Space Data Systems
CFA	Colour Filter Array
CPU	Central Processing Unit
CR	Compression Ratio
DPCM	Differential Pulse-Code Modulation
FPS	Frames Per Second
FPGA	Field-Programmable Gate Array
GPU	Graphics Processing Unit
IP core	Intellectual Property core
LVDS	Low-Voltage Differential Signalling
VHDL	VHSIC Hardware Description Language
VHSIC	Very High Speed Integrated Circuit
YCCC	Y-Cd-Cm-Co Colour Space
